# The Emerging Short Chain Fatty Acid Enriched Metabotype in Irritable Bowel Syndrome and Its Potential Clinical Relevance

**DOI:** 10.1111/apt.70677

**Published:** 2026-04-17

**Authors:** T. E. Conley, A. Duncan, A. Modasia, A. C. Ford, D. M. Pritchard, F. Hildebrand, F. J. Warren, R. Spiller, C. S. Probert

**Affiliations:** ^1^ Institute of Systems Molecular and Integrative Biology University of Liverpool Liverpool UK; ^2^ Department of Gastroenterology Liverpool University Foundation NHS Trust Liverpool UK; ^3^ Quadram Institute Bioscience Norwich Research Park Norwich UK; ^4^ Earlham Institute Norwich Research Park Norfolk UK; ^5^ Leeds Gastroenterology Institute Leeds Teaching Hospitals NHS Trust Leeds UK; ^6^ Leeds Institute of Medical Research at St. James's University of Leeds Leeds UK; ^7^ School of Biological Sciences University of East Anglia Norwich UK; ^8^ Nottingham Digestive Diseases Centre Nottingham University Hospitals NHS Trust Nottingham UK; ^9^ NIHR Nottingham Digestive Diseases Biomedical Research Centre University of Nottingham Nottingham UK

**Keywords:** irritable bowel syndrome, metabolome, metabotype, volatile organic compounds

## Abstract

**Background:**

Metabolomic analysis in irritable bowel syndrome (IBS) has identified metabotypes enriched in faecal short‐chain fatty acids (SCFAs), but it remains unclear whether this reflects rapid colonic transit or if these metabolites actively contribute to pathophysiology.

**Aims:**

We aimed to determine whether an SCFA metabotype could be identified within a cohort of patients with moderate–severe IBS‐D and assess whether this metabotype associated with greater clinical severity, alterations in gut transit time and specific microbiome features.

**Methods:**

This was a post hoc cross‐sectional exploratory analysis of baseline data from the multicentre, randomised, placebo‐controlled trial of ondansetron in IBS‐D (TRITON: ISRCTN17508514). Faecal volatile organic compounds were profiled by GC–MS. The microbiome was characterised by whole‐genome shotgun metagenomic sequencing. Unsupervised hierarchical clustering was used to identify an SCFA‐enriched metabotype and non‐negative matrix factorisation (NMF) enabled the derivation of complementary metabosignatures by assessing continuous gradients in metabolite composition.

**Results:**

A SCFA‐enriched metabotype was identified in 20/63 participants (31.7%). This metabotype was associated with more severe abdominal pain, urgency, increased stool frequency and faster whole‐gut transit. NMF identified three metabosignatures: S3 was typified by a high proportion of SCFAs and captured the SCFA‐enriched metabotype, while S1 and S2 corresponded to the non‐SCFA (“Other”) metabotype. SCFA relative abundance positively correlated with symptom severity and inversely correlated with transit time. The Other metabotype and S1/S2 signatures were enriched in taxa linked to slower transit, whereas S3 showed no overlapping taxa with the SCFA metabotype.

**Conclusion:**

A faecal metabotype enriched in SCFAs associated with an IBS‐D phenotype characterised by pain, urgency, rapid transit and higher stool frequency.

## Graphical abstract

In a cohort of IBS‐D patients recruited to the TRITON study, faecal metabolomic profiling reveals a SCFA‐enriched metabotype associated with increased pain and urgency, altered stool characteristics and accelerated intestinal transit.

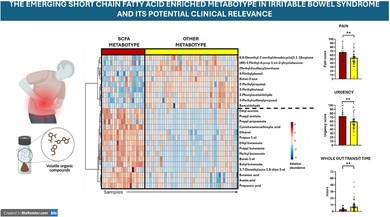

## 1 Introduction

Irritable bowel syndrome (IBS) impacts an estimated 4% of the global population [1] and in the United Kingdom direct healthcare costs associated with IBS are estimated to exceed £1.3 billion per annum [2]. Nevertheless, despite its high prevalence and substantial healthcare burden, the pathophysiology of IBS remains incompletely understood, underscored by the fact that the underlying causes of abdominal pain and visceral hypersensitivity in IBS are ranked among the Top 10 research priorities in the field [3].

The analysis of faecal volatile organic compounds (VOCs) provides a fast, cost‐effective way to study the functional gut microenvironment. The human volatome includes nearly 3000 VOCs, of which 443 have been identified in faecal samples [4]. Many of these VOCs are largely by‐products of colonic microbial metabolism, although some are derived directly from diet and from host epithelial metabolism. When studying the microbial communities (comprising bacteria, archaea, viruses and fungi) that contribute to the gut microbiome, emerging evidence suggests that microbial function, rather than community composition, is more closely linked to clinical outcomes [5]. Therefore, integrating faecal metabolomics with microbial analysis is important to understand the pathophysiological mechanisms underlying IBS. To date, there have been few published studies on faecal VOCs in IBS [6, 7, 8, 9, 10, 11, 12, 13, 14].

Metabolomic analysis in IBS has hinted towards the presence of a faecal metabotype enriched in short‐chain fatty acids (SCFAs) [13, 14, 15, 16, 17], although consistent, robust microbial–metabolite signatures remain elusive, reflecting heterogeneity in study design and limited sample sizes. SCFAs are essential for gastrointestinal (GI) health [18], but their effects may not be universally beneficial. Indeed, SCFAs may exert pro‐nociceptive or pro‐kinetic effects depending on their concentration, site of production and the surrounding colonic milieu [19, 20, 21]. Whether the presence of this SCFA‐rich faecal metabotype simply reflects an IBS phenotype associated with faster colonic transit or whether it denotes a more pathophysiologically distinct and potentially severe IBS subtype is unclear.

SCFA production by the gut microbiome is influenced by several factors, including diet, medications and lifestyle factors [14, 22, 23, 24]. A major factor influencing gut microbiome composition appears to be whole gut transit time (WGTT), which is closely linked to stool consistency [24, 25, 26, 27, 28]. Rapid WGTT is associated with greater abundance of faster‐growing bacterial species such as *Faecalibacterium prausnitzii* and *Bacteroides* species, which are the main SCFA producers in the human gut microbiome, while slow WGTT is associated with slow‐growing species such as *Akkermansia municiphillia* and the archaea *Methanobrevibacter smithii* [26]. Rapid transit in IBS‐D patients has been associated with elevated SCFA, although the link to microbiome composition is complex and may involve other factors such as bile acids [29].

It is hypothesised that a degree of ‘metabolic virulence’ could underpin symptom expression in IBS [22]. Indeed, a metabotype rich in SCFAs might not necessarily result in a more severe IBS phenotype as a direct consequence of these metabolites. Instead, it might be a marker of a broader, more complex IBS microenvironment mediating symptoms through stimulation of a cascade of more complex downstream neuroactive metabolites [30]. Conversely, the absence of an SCFA‐rich metabotype might be protective, potentially reducing symptom severity [14].

In this study, we investigated whether a distinct baseline SCFA metabotype could be identified in a cohort of patients who had IBS with diarrhoea (IBS‐D), recruited from multiple sites across the United Kingdom. Additionally, we examined the hypothesis that this metabotype enriched in SCFAs is associated with a more severe IBS phenotype and explored potential relationships between specific SCFA metabolites and key IBS symptoms.

## 2 Methods

This was a post hoc cross‐sectional exploratory analysis using clinical data and baseline stool samples collected and curated as part of the TRITON study [31], a phase III, parallel group, randomised, double‐blind, placebo‐controlled trial investigating the effect of the 5‐HT3 receptor antagonist, ondansetron, on symptom severity in IBS‐D. Ethical approval was granted by the Yorkshire and the Humber Leeds West REC (reference 17/YH/0262). All authors had access to the study data and reviewed and approved the final manuscript.

### 2.1 Study Design

Briefly, adult participants were recruited from 13 UK centres from 2018 to 2020 (Supporting Informations 1). Cases were defined by the fulfilment of the Rome IV criteria for IBS‐D. Inclusion and exclusion criteria are summarised in Supporting Informations 2. Clinical and mechanistic data were collected alongside the collection of biological material (Table 1). The mechanistic metric, WGTT, was measured using radio‐opaque markers, modified from Metcalf et al. [32] as previously described [31]. The faeces collection process is outlined in Supporting Informations 3.

**TABLE 1:** IBS metrics used.

IBS metrics	Measurement tool	Potential score
IBS symptom severity	IBS symptom severity scale	175–500
Pain	100‐point visual analogue scale	30–100
Urgency	100‐point visual analogue scale	−/100
Stool form	Bristol stool form scale	1–7
Stool frequency	Numeric	Numeric
Loose stool days^a^	Numeric	Numeric
Depression	HADS depression sub score	−/21
Anxiety	HADS anxiety sub score	−/21
Whole gut transit time	Radio‐opaque markers and an abdominal radiograph	Numeric (h)

Abbreviations: HADS, hospital anxiety and depression score; IBS‐SSS, IBS symptom severity score.

^a^ Number of days per week with at least one stool BSFS > 5.

### 2.2 Sample Processing

#### 2.2.1 Solid Phase Micro‐Extraction Gas Chromatography Mass Spectrometry

An optimised method described by Reade et al. was utilised throughout [33]. One hundred milligrammes aliquots of stool were stored in 10 mL headspace vials (Supelco, Germany) at −80°C until analysis. Aliquoting was performed in a Class II microbiological safety cabinet.

### 2.3 Faecal Headspace Volatile Organic Compound Analysis

Gas chromatography mass spectrometry (GC–MS) was used to examine the faecal headspace. Prior to GC–MS analysis VOCs were extracted from the headspace using a DVB‐CAR‐PDMS 50/30 μm solid phase micro‐extraction (SPME) fibre (Sigma‐Aldrich, UK). The SPME‐GCMS pipeline used a Combi PAL auto‐sampler (CTC Analytics, Switzerland) and a PerkinElmer Clarus 500 GC–MS quadrupole benchtop system (PerkinElmer, Beaconsfield, UK).

### 2.4 DNA Extraction and Metagenomic Sequencing

DNA was extracted from frozen faecal samples using a Promega Maxwell RSC 48 Instrument automated nucleic acid purification system with the Maxwell RSC Faecal Microbiome DNA Kit. DNA was extracted from ~200 mg faecal samples (sample homogenisation in FastPrep 3 × 60 s at speed 6.0), following the manufacturer's instructions. Library preparation was carried out using a modified version of the Illumina Nextera DNA Flex Library Prep Kit, as described by Costigan et al. [27]. Metagenomic sequencing was carried out using three 10B lanes of an Illumina NovaSeq X Plus instrument by Novogene (Cambridge, UK). This yielded an average of 10 GB of data per sample.

### 2.5 Data Processing

#### 2.5.1 VOCs

GC–MS data were processed using a three‐step pipeline, utilising: (i) the Automated Mass Spectral Deconvolution and Identification System software (AMDIS, Version 2.73, 2017) for chromatogram deconvolution and VOC identification, with settings optimised for faecal VOCs detection [33]; (ii) the NIST Mass Spectral Library and Search Program (Version 2.3, 2017) for compound assignment; and (iii) the R package Metab (Version 4.2.2, 2022) for retention time alignment and relative abundance quantification. AMDIS and NIST were used to curate an in‐house VOC library, with compound assignments detailed in Supporting Informations 4 and named according to International Union of Pure and Applied Chemistry (IUPAC) nomenclature.

#### 2.5.2 Bioinformatic Analysis

The raw data were processed using the MG‐TK pipeline (formerly MATAFILER) [34]. Specifically, reads were filtered based on quality using sdm [35]. Reads matching the human genome were identified with kraken2 and filtered out. Samples from the same individual were co‐assembled using MEGAHIT and binned using SemiBin. Reads were mapped to the assembly with Bowtie 2 and counted among samples using rtk [36]. Genes were predicted with Prodigal and SNP calling was performed using bcftools. MAGs were clustered into metagenomic species (MGS) and then taxonomically profiled based on marker genes using GTDB‐tk [37] and LCA algorithm [35].

### 2.6 Statistical Analysis

#### 2.6.1 VOCs

All statistical analyses were performed using ‘R’ (Version 4.2.2, 2022) and IBM SPSS Statistics 24 (Version 28.0.1.1). VOC abundance data analysis was undertaken using the online platform, MetaboAnalyst 5.0 (Canada, Version 12.0).

VOC abundance data were subject to pre‐processing prior to statistical analysis. First, the dataset was filtered to ensure statistical models were robust; variables were excluded when more than 50% of the data were missing in each biological group. Below this threshold, missing data values were replaced by imputation with 20% of the lower limit of detection in the dataset for each VOC concerned. Relative abundance data were normalised according to their median value, log_10_‐transformed and auto‐scaled (mean‐centred and divided by the standard deviation of each variable). The distribution of VOC abundance data was assessed for normality by Shapiro–Wilk test and visual inspection of histograms/Q–Q plots. Univariate analysis used Mann–Whitney *U* tests where data followed a non‐parametric distribution, otherwise *t*‐tests were used.

After data pre‐processing, the 50 remaining VOCs were carried forward into the statistical analysis. The SCFA metabotype was identified by hierarchical clustering (Ward's linkage, Euclidean distance) and confirmed on principal component analysis. The clusters were observed on the unsupervised heatmap, without any predefined labels. Next, the clusters were categorised as either the ‘SCFA metabotype’ or the ‘Other metabotype’ based on their metabolic signature. To confirm these two metabotypes, a Self‐Organising Map (SOM) was generated and trained using Euclidean distance and a Gaussian neighbourhood function (Supporting Informations 5) and the composition of VOCs decomposed to metabosignatures using non‐negative matrix factorisation (NMF). Following this classification, the dataset was re‐analysed to explore differences and patterns according to these newly defined groups.

#### 2.6.2 Microbiome Associations

Associations between microbiome composition and clinical metadata were undertaken using mixed effects linear modelling using an FDR‐adjusted *p*‐value cut‐off of 0.05 for significance in the r package Microviz [38]. Data were filtered to exclude taxa at below 10% prevalence prior to analysis. Microbial associations with both the metabotypes and metabosignatures were analysed using the linear discriminant analysis (LDA) effect size (LEfSe) method [39] with *p*‐value cut‐off set to 0.05 and an LDA cut‐off of 3.

#### 2.6.3 Clinical Metrics

Demographic data were compared after re‐allocation according to these new groups (using an independent samples *t*‐test and *χ* ^2^ testing) to ensure potential confounders were identified before forward analysis.

Baseline and mechanistic data were compared between the two groups using an independent samples *t*‐test and *χ* ^2^ testing. Pearson's correlation coefficients were calculated to detect any relationships between clinical symptom metrics, WGTT and VOC abundance in this cohort. A ‘Min‐Max scaler’ was employed to normalise symptom score data with VOC relative abundance data. This process rescaled both datasets to a common range, ensuring comparability and mitigation of the impact of differing units and value ranges.

## 3 Results

**FIGURE 1:**
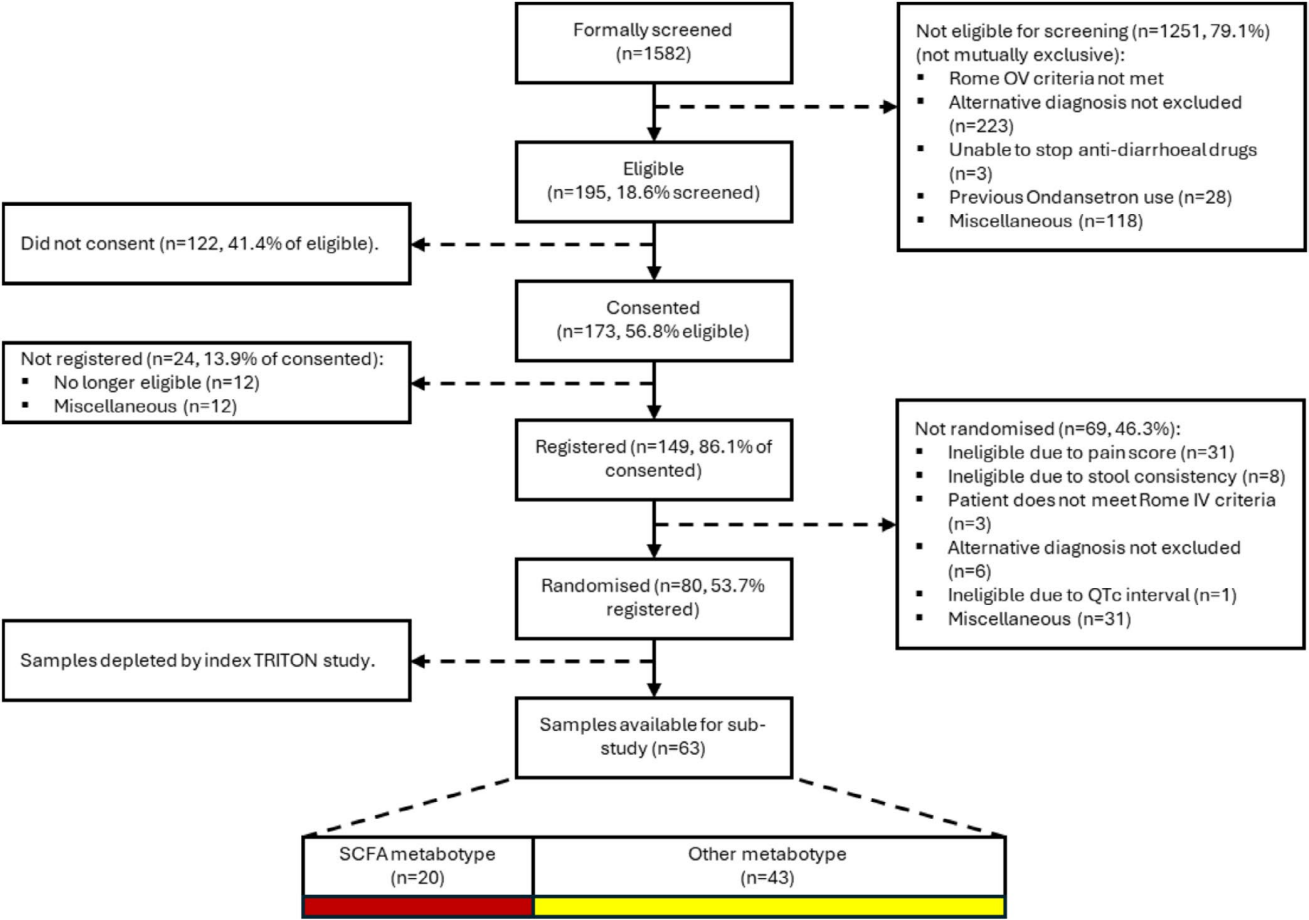
PRISMA chart adapted from the index TRITON study.

Baseline faecal samples from 63 TRITON participants were analysed (Figure 1, Table 2). The SCFA metabotype was exhibited by 32% of the study population (*n* = 20). Of the 50 VOCs analysed, 31 were identified as contributing significantly to group membership at baseline, with significance defined by an FDR‐adjusted *p*‐value of < 0.1. Sixteen VOCs were present in significantly higher relative abundance in the SCFA metabotype group and 15 VOCs were present in significantly higher relative abundance in the Other metabotype group (Table 3). The Other metabotype was characterised by a relative deficiency of SCFAs and associated metabolites and a relative excess of ketones, aldehydes, terpenes and organosulphurs.

**TABLE 2:** Demographic distribution of study participants after re‐allocation according to metabotype.

IBS metric	SCFA metabotype (*n* = 20)	Other metabotype (*n* = 43)	*p*
Case number	20	43	—
Age (mean ± SD)	42.1 (±15.7)	44.9 (±15.9)	ns^a^
Age distribution (%)			
< 40 (%)	9 (45.0)	20 (46.5)	ns^a^
40–59 (%)	9 (45.0)	14 (32.6)	ns^a^
> 60 (%)	2 (10.0)	9 (20.9)	ns^a^
Female (%)	12 (60.0)	25 (58.1)	ns^b^
IBS‐SSS (mean ± SD)	379.40 ± 73.1	340.9 ± 84.5	ns (0.073)^a^
IBS‐SSS distribution			
175–299 (%)	3 (15.0)	11 (25.6)	ns^a^
300–500 (%)	17 (85.0)	32 (74.4)	ns^a^
Abdominal pain (mean ± SD)	67.14 ± 17.4	53.10 ± 16.6	< 0.01^a^
Urgency (mean ± SD)	72.68 ± 19.0	59.03 ± 16.6	< 0.01^a^
Stool frequency (mean ± SD)	5.19 ± 2.5	3.53 ± 1.5	< 0.05^a^
Stool form (mean ± SD)	5.72 ± 0.8	5.19 ± 0.7	< 0.05^a^
Loose stool days (mean ± SD)	6.15 ± 1.3	5.38 ± 1.2	< 0.05^a^
Anxiety (mean ± SD)	9.55 ± 5.2	9.58 ± 4.7	ns^a^
Depression (mean ± SD)	7.85 ± 3.5	6.61 ± 4.0	ns^a^
WGTT (median, IQR)	3.60 (4.8)	8.40 (8.4)	< 0.01^**c**^

Abbreviations: IBS‐SSS, irritable bowel syndrome severity scoring system; ns, non‐significant; SD, standard deviation.

^a^ Independent samples *t*‐test.

^b^ *χ* ^2^ test.

^c^ Mann–Whitney *U* test.

**TABLE 3:** Volatile organic compounds demonstrating significant differences in relative abundance between metabotypes.

Retention time (min)	Volatile organic compound	Family	Unadjusted *p*	*q* (FDR)
*VOCs abundant in SCFA metabotye*				
20.13	Butanoic acid	SCFA	< 0.0001	< 0.0001
32.04	Cyclohexanecarboxylic acid	CHC	< 0.0001	< 0.0001
7.10	Ethanol	Primary alcohol	< 0.0001	< 0.0001
10.10	Propan‐1‐ol	Primary alcohol	< 0.0001	< 0.0001
13.36	Acetic acid	SCFA	< 0.0001	< 0.0001
15.14	Methyl butanoate	SCFA‐ester	< 0.0001	< 0.0001
21.86	Propyl butanoate	SCFA‐ester	< 0.0001	< 0.0001
16.89	Propanoic acid	SCFA	< 0.0001	< 0.0001
18.12	Ethyl butanoate	SCFA‐ester	< 0.0001	< 0.0001
14.00	Butan‐1‐ol	Primary alcohol	< 0.0001	< 0.0001
14.83	Propyl acetate	SCFA‐ester	< 0.0001	< 0.0001
11.08	Ethyl acetate	SCFA‐ester	< 0.0001	< 0.0001
25.44	Butyl butanoate	SCFA‐ester	< 0.001	< 0.001
18.49	Propyl propanoate	SCFA‐ester	< 0.001	< 0.001
29.72	3,7‐dimethylocta‐1,6‐dien‐3‐ol	Monoterpenoid alcohol	< 0.001	< 0.001
23.66	Pentanoic acid	SCFA	< 0.001	< 0.001
*VOCs abundant in other metabotype*				
31.35	4‐Methylphenol	Phenol	< 0.0001	< 0.0001
24.11	3‐Methylsulfanylpropanal	Aldehyde	< 0.0001	< 0.0001
26.14	Benzaldehyde	Aldehyde	< 0.0001	< 0.0001
13.10	3‐Methylbutanal	Aldehyde	< 0.0001	< 0.0001
9.55	2‐Methylpropanal	Aldehyde	< 0.0001	< 0.0001
29.14	2‐Phenylacetaldehyde	Aldehyde	< 0.0001	< 0.0001
16.55	(Methyldisulfanyl)methane	Organosulphur	< 0.0001	< 0.0001
26.56	(4R)‐1‐Methyl‐4‐prop‐1‐en‐2‐ylcyclohexene	Terpene	< 0.001	< 0.001
24.76	6,6‐dimethyl‐2‐methylidenebicyclo[3.1.1]heptane	Terpene	< 0.01	< 0.01
11.09	Butan‐2‐one	Ketone	< 0.01	< 0.05
29.89	Nonanal	Aldehyde	< 0.05	< 0.05
26.00	6‐Methylhept‐5‐en‐2‐one	Ketone	< 0.05	< 0.1
24.76	7‐Methyl‐3‐methylideneocta‐1,6‐diene	Terpene	< 0.05	< 0.1
26.69	1‐Methyl‐4‐propan‐2‐ylbenzene	Terpene	< 0.1	< 0.1
39.01	1H‐Indole	Indole	< 0.1	< 0.1

Abbreviations: CHC, cyclohexanecarboxylic acid; FDR, false discovery rate; SCFA, short chain fatty acid.

**FIGURE 2:**
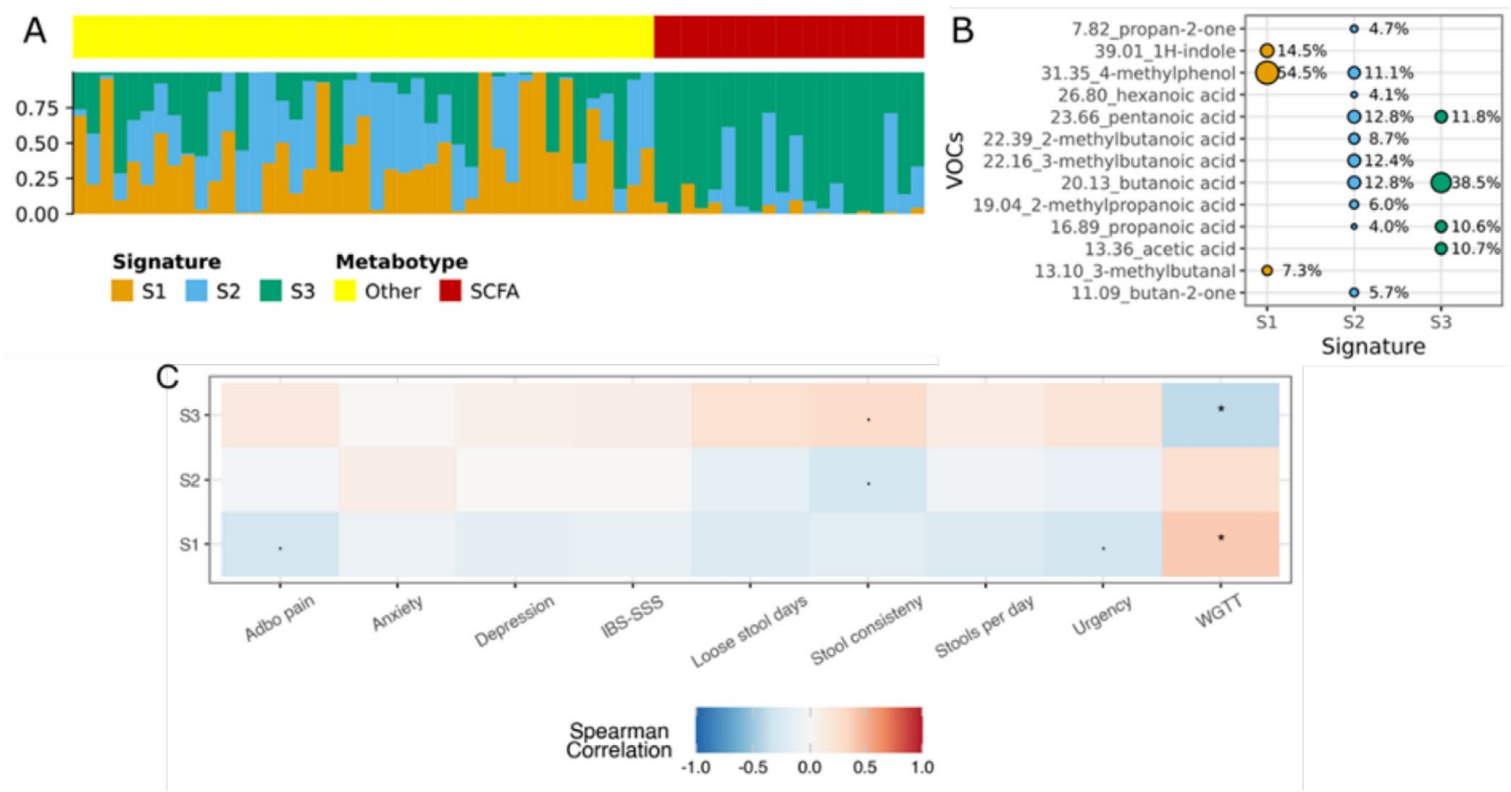
Metabosignatures and their association with symptoms. (A) Metabosignature profiles for individual participants. The metabotype of each participant is indicated in the bar, with red representing the SCFA metabotype and yellow representing the other metabotype. (B) The contribution of individual VOCs to each of the metabotypes. (C) Spearman rank correlations between metabosignatures and clinical symptoms. Associations with a BH adjusted *p*‐value < 0.05 are indicated with stars, while associations with an BH adjusted *p*‐value < 0.1 are indicated with points.

Three signatures identified using NMF were in concordance with the metabotype clusters: samples in the SCFA metabotype have a high abundance of signature S3, which is typified by a high proportion of SCFAs. Signature S2 contains a lower proportion of SCFAs alongside branched SCFAs and is found in a higher weight in samples in the Other metabotype. Signature S1 is also found at a higher weight in the Other metabotype and is characterised by indole and methylphenol (Figure 2).

### 3.1 The Impact of Metabotype on IBS Symptoms

**FIGURE 3:**
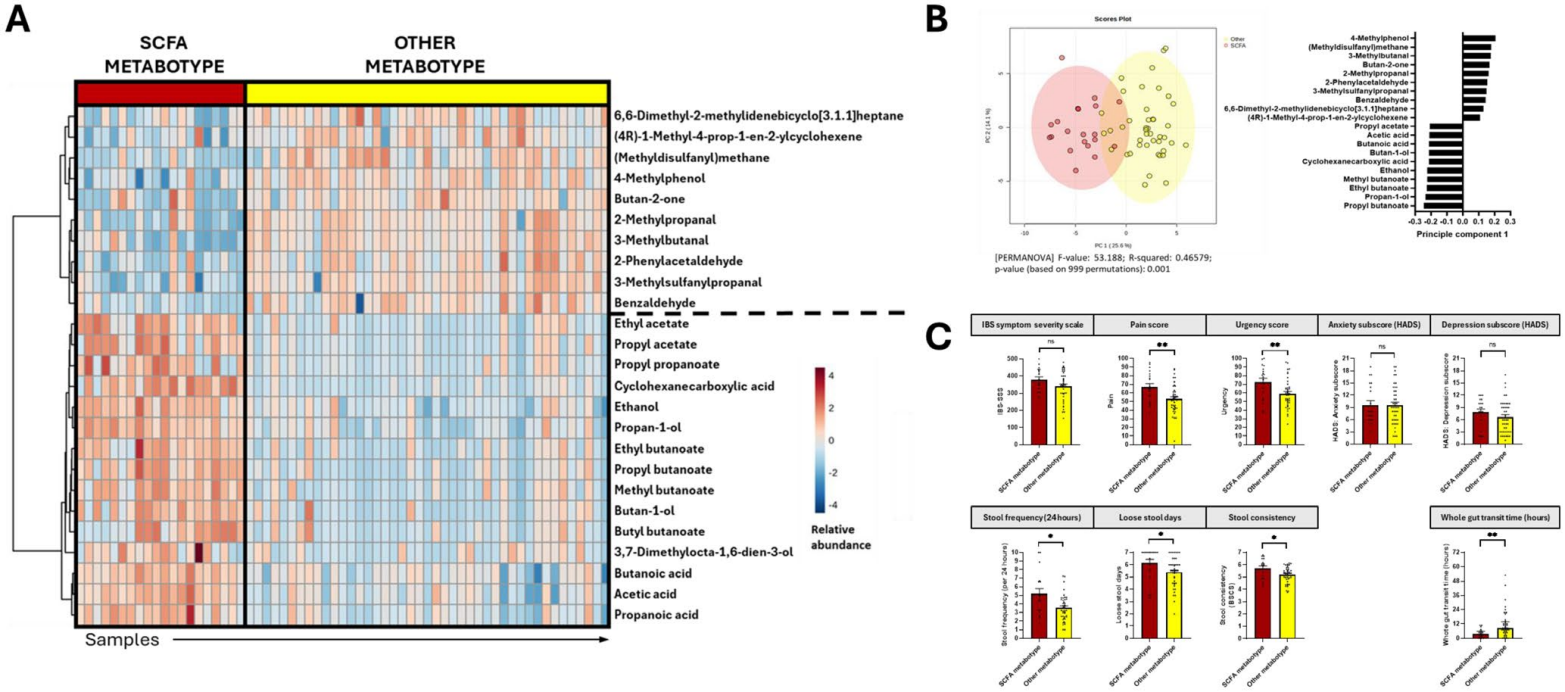
(A) Metabolite (VOC) heatmap hierarchically clustered to identify potential metabotypes. The top 25 VOCs (those with the most significant *p* values on univariate analysis with Wilcoxon Rank Sum test) were used to generate this heatmap. The short‐chain fatty acid (SCFA) metabotype was unmasked using this approach. (B) Left: Principal component analysis demonstrating clear separation of the two metabotypes at baseline; Right: The top 10 VOCs contributing to the positive and negative loading scores among principal component 1 (PC1). (C) Differences in symptom profiles between metabotypes. Independent samples *t*‐test used for all comparisons except for whole gut transit time (WGTT)—Mann–Whitney *U* test. Error bars depict standard error of mean (WGTT error bars depict IQR). *p < 0.05; **p < 0.01; ns, non‐significant. IBS‐SSS, IBS symptom severity score.

Individuals with the SCFA metabotype were characterised by significantly higher abdominal pain scores, urgency scores, greater stool frequency, looser stool form and an increased frequency of ‘loose stool days’ (Figure 3). WGTT was significantly faster in those with the SCFA metabotype. No significant differences were identified in anxiety or depression symptom scores.

### 3.2 Symptom‐Symptom Correlation Analysis

**FIGURE 4:**
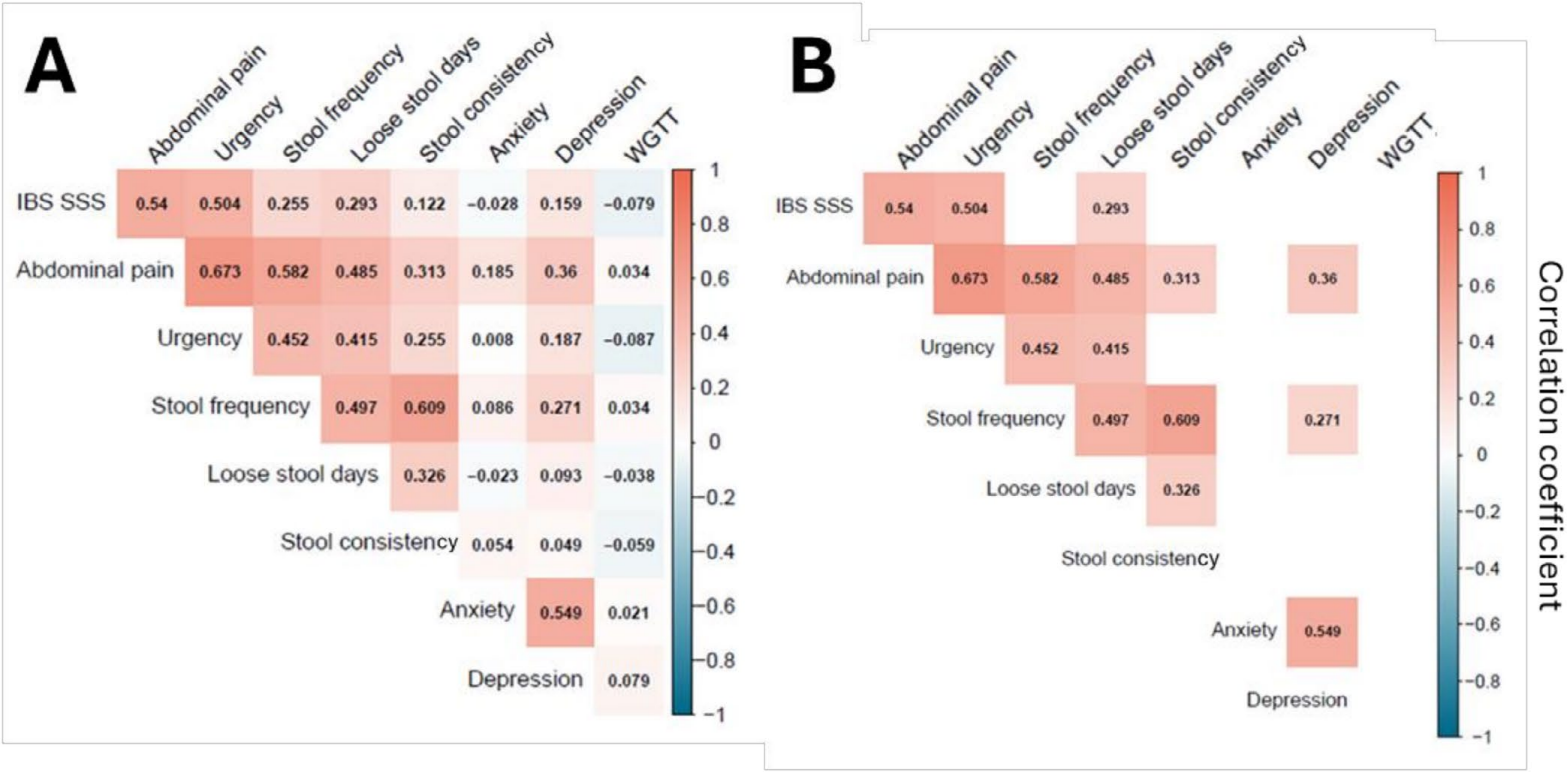
Correlation plot illustrating Pearson's correlation coefficients and the intra‐symptom relationships observed in this cohort of patients with IBS‐D. (A) All correlation coefficients irrespective of *p* value. (B) Same correlation plot with correlation coefficients removed if *p* < 0.05.

Pearson's correlation coefficients were calculated to determine the degree of association between different IBS metrics (Figure 4). A strong positive correlation was observed between symptom metrics, for example abdominal pain and urgency (*r* = 0.673). Stool consistency/form correlated strongly with stool frequency (*r* = 0.609). No significant correlations were found between any IBS metric and WGTT.

### 3.3 Metabolite‐Metabolite Correlation Relationships

**FIGURE 5:**
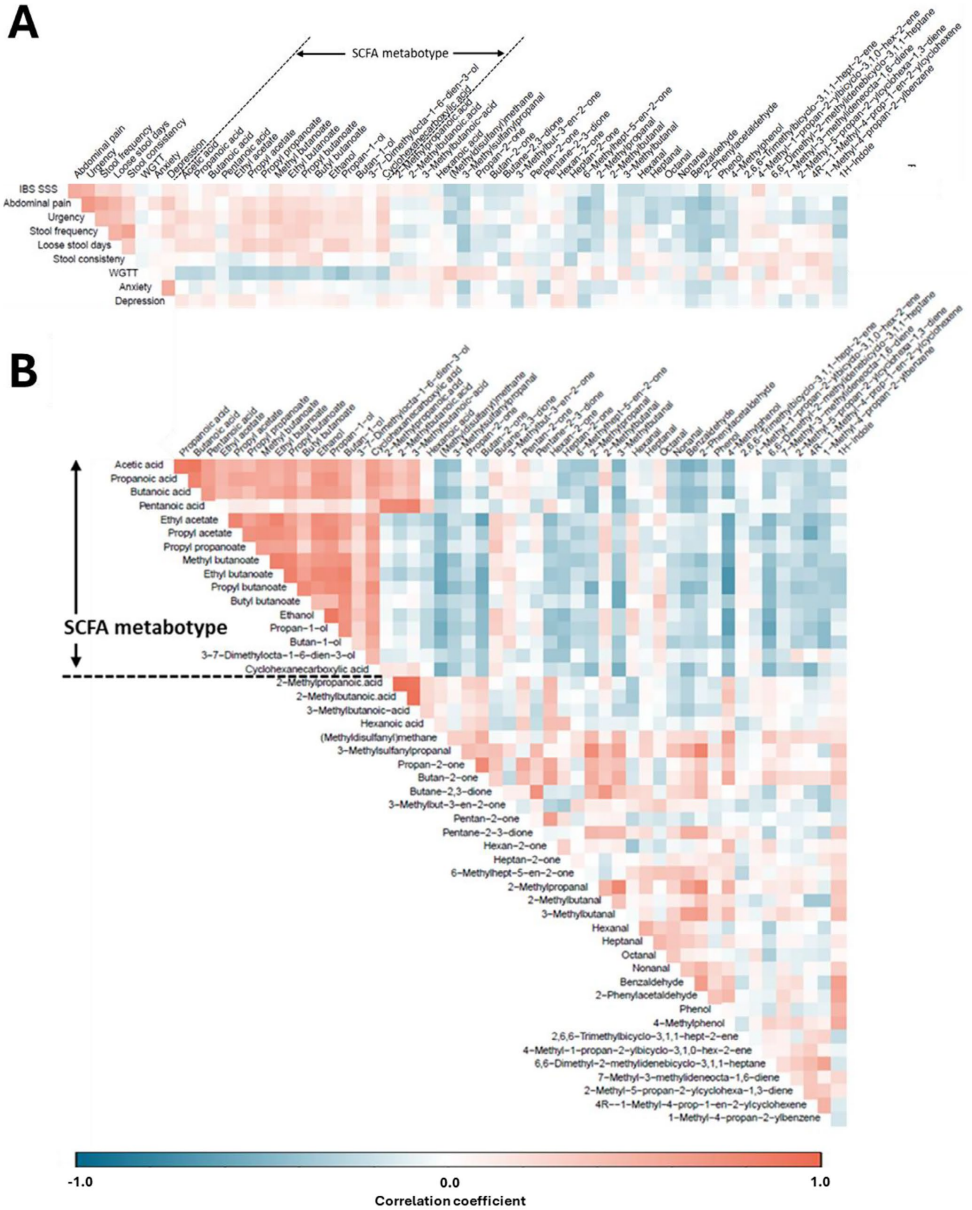
(A) Correlation plot demonstrating the pattern of Pearson's correlation coefficients between all 50 volatile organic compounds included in the analysis and the IBS metrics measured. (B) Correlation plot illustrating the correlation relationships between volatile organic compounds (VOCs). The top left‐hand corner of the plot contains the compounds characterising the short chain fatty acid metabotype. Note how these VOCs correlate positively with metabolites in the same group, while the inverse is observed when considering their relationship with VOCs characterising the ‘other’ metabotype.

Metabolite‐metabolite correlation analysis demonstrated clear clustering both within metabolite families (e.g., alcohols correlating with alcohols and aldehydes correlating with aldehydes) and within the two metabotypes (e.g., SCFAs correlating with alcohols [both members of the SCFA metabotype] and ketones correlating with aldehydes [both members of the Other metabotype]) (Figure 5).

Positive metabolite–metabolite correlation coefficients were generally less strong within the ‘Other metabotype’, perhaps suggesting that this group is less clear cut and are as much defined by their negative correlation with SCFAs and SCFA‐associated metabolites as they are by their positive correlation relationship with metabolites within their cluster. Indeed, the unsupervised metabosignature analysis highlighted that the Other metabotype may be composed of two separate metabosignatures.

### 3.4 Associations Between Metabolites and Symptoms

Correlation analyses were performed to examine the relationship between metabolites and IBS metrics. Correlation coefficients were calculated to determine the relationship between IBS metrics and the 31 discriminatory VOCs characterising metabotype (Supporting Informations 6). Clear differences in IBS behaviour were noted between the two metabotypes. The relative abundance of metabolites characterising the SCFA metabotype correlated positively with symptom severity on the IBS‐SSS, pain, urgency and stool frequency and negatively with WGTT (Figure 5). Conversely, the metabolites characterising the ‘Other’ group (ketones, aldehydes and organosulphurs) negatively correlated with IBS‐SSS sub‐scores and symptom metrics, while positively correlating with transit time.

Metabosignature S3, which has a high proportion of SCFAs, showed a negative Spearman's correlation to WGTT (*r* = −0.39, *p* = 0.035, BH adjustment), while the other signatures S1 and S2 showed a positive correlation to WGTT (*r* = 0.40, 0.27, *p* = 0.001, 0.0035 respectively, BH adjustment). The metabosignatures also showed weaker associations with urgency, stool consistency and abdominal pain.

### 3.5 Associations Between Metabotypes and the Microbiome

**FIGURE 6:**
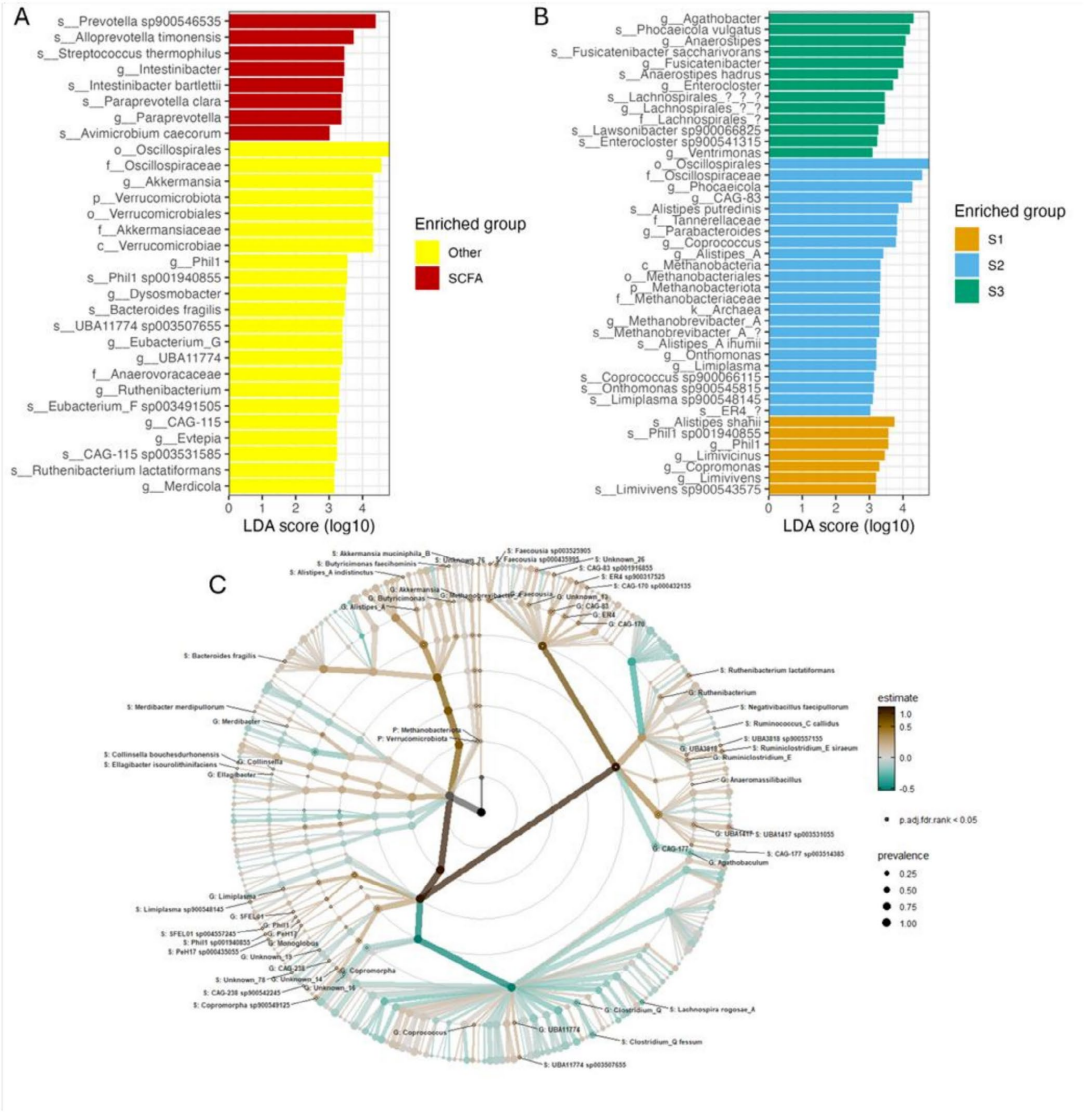
Microbiome associations with metabotype, metabosignatures and WGTT. (A) Microbiome associations with metabotype determined by lefse with a *p*‐value cut‐off of 0.05 and an LDA cut‐off of 3. (B) Microbiome associations with metabosignature determined by lefse with a *p*‐value cut‐off of 0.05 and an LDA cut‐off of 3. (C) Cladeogram showing microbiome associations with WGTT. The colour indicates the association between taxa and WGTT. The node size indicates the prevalence of the taxa. Significant associations, following a FDR corrected *p*‐value cut‐off of 0.05 are indicated as point, with names given at genus and species level.

Several microbial taxa associated with both the metabotypes and metabosignatures (Figure 6). At family level, an overlap was observed between the taxa associated with the Other metabotype and the S2 metabosignatures, with Oscillispiraceae being enriched in both. No overlapping taxa were observed between the S3 metabosignature and the SCFA metabotype. Interestingly, we observed several taxa associated with slower WGTT were also associated with the Other metabotype and with the S1/S2 metabosignatures. The species *Ruthenibacterium lactatiformis, UBA11774 sp003507655* and *Bacteroides fragilis* and the genus *Akkermansia* were enriched in the Other metabotype and were associated with slower WGTT. The genera *Methanobrevibacter_A, Alistipes_A, CAG‐83, Coprococcus* and *Limiplasma* were associated with slower WGTT and the S2 metabosignature, while the genus *Phil1* was associated with slower WGTT and the S1 metabosignature.

## 4 Discussion

These findings lend support to the hypothesis that a baseline faecal metabotype enriched in SCFAs exists in IBS, is clinically relevant and hint towards the potential link between microbially derived metabolites and symptoms. We further present a novel metabosignature model which describes gradients in the faecal metabolome composition rather than characterisation by discrete clusters, enabling the incorporation of objective biological data into what has traditionally been a symptom‐driven and clinically heterogeneous disorder. In this context, the identification of reproducible, biologically meaningful subgroups—such as the SCFA‐enriched metabotype described here—may help bridge the gap between symptoms and underlying pathophysiology and has the potential to refine patient stratification and support more personalised, biologically‐informed management strategies.

When undigested complex carbohydrates reach the colon they are metabolised by the gut microbiota to SCFAs and other organic and amino acids [40]. However, faecal SCFAs represent only a small fraction of total colonic SCFA production and instead reflect the integrated effects of dietary substrate availability, microbial fermentative capacity and host factors such as epithelial absorption and GI transit time [41]. Variation in these determinants can substantially reshape microbial activity and the gut microenvironment, influencing metabolite profiles.

While faecal SCFAs are positively associated with accelerated GI transit in IBS‐D [29, 42], it remains uncertain whether their increased faecal abundance reflects a downstream effect of more rapid transit or whether SCFAs themselves contribute to transit acceleration. It is likely that both mechanisms co‐exist. Experimental data support this bidirectional relationship: colonic infusion of acetic acid reduces transit time in murine models [43], possibly implicating SCFA imbalance in the pathogenesis of non‐constipated IBS, while pharmacologically induced acceleration of transit has been convincingly shown to increase faecal SCFA concentrations in humans [41, 44]. In contrast, slower GI transit is associated with reduced faecal SCFA levels, with faecal propanoic acid and butanoic acid demonstrating negative correlations with transit time in individuals with slow transit constipation [45]. Collectively, these findings emphasise that faecal SCFA profiles reflect the integrated effects of transit, microbial production/fermentation and host absorption. Within this framework, the identification of faecal metabotypes provides a pragmatic means of distinguishing biologically distinct patient groups with potential clinical relevance.

The microbiome findings in this study are exploratory and intended to describe taxa associated with the identified metabotypes rather than to establish mechanistic pathways. There is a well‐established association between transit time and microbiome composition [26, 28] and the observed patterns largely reflected known transit‐related microbial ecologies, supporting ‐ but not proving ‐ a link between colonic metabolic environment and symptom phenotype. In this study we demonstrate that a similar group of microbes is also associated with metabolite profiles. Rapidly growing, saccharolytic species were found to be associated with the SCFA metabotype and the S3 metabosignature, while slower growing species such as *A. municiphilia* and *M. smithii* were associated with the Other metabotype and the S1/S2 metabosignature. Previous studies have highlighted a strong correlation between these species and stool SCFA levels [46] further indicating the complex relationship between stool microbiome and metabolome. Notably, no individual taxa overlapped between those identified as associated with the S3 metabosignature and the SCFA metabotype, despite both reflecting an SCFA‐enriched metabolic environment. This finding highlights the complex and potentially many‐to‐one relationship between microbial composition and metabolic output, consistent with functional redundancy within the gut microbiome.

Obviously, faster colonic transit times correlate with increased stool frequency and more‐liquid stool form. However, faster transit does not reliably associate with symptom severity or pain in IBS [47]. Our findings support this lack of association and add support to the notion that microbially derived gut metabolites mediate symptoms in patients with IBS‐D. Indeed, Vervier et al. in their case–control study comprised of non‐constipated IBS subjects demonstrated that those with a colonic microenvironment bacterially primed to produce SCFAs not only experienced more severe symptoms at baseline but demonstrated higher magnitude symptom improvement following FODMAP restriction (in line with higher magnitude reductions in SCFA concentration) [22]. Moreover, in their study SCFA metabolites correlated strongly with bacterial tryptophan synthesis pathways, whereas metabolites defining our Other metabotype, such as aldehydes and ketones, showed inverse correlations, potentially reflecting differences in the generation of unmeasured downstream neuroactive mediators [14].

Our findings show that a faecal SCFA‐enriched metabotype is associated with greater pain, urgency and more severe stool metrics in IBS‐D, suggesting that SCFAs may be relevant to symptom expression and warranting closer examination of how specific SCFA metabolites could contribute to visceral hypersensitivity in IBS. However, the present study cannot determine whether these metabolites actively drive symptoms or simply reflect a transit‐related microenvironment characteristic of more symptomatic patients. Early work by Tana et al. demonstrated that higher faecal concentrations of propanoic and acetic acid were linked to increased GI symptom burden in IBS patients [48] and several experimental models have demonstrated potential pro‐nociceptive effects of specific SCFAs, including direct stimulation of afferent neurons [49] and the modulation of downstream mediators such as serotonin [50, 51, 52, 53]. Together, these observations suggest that SCFAs may contribute to symptom generation in at least a subset of individuals with IBS, but our data remain associative. More work is needed to clarify the mechanistic relevance of these metabolites, particularly as previous studies indicate that some IBS‐D patients may be uniquely susceptible to serotonergic‐driven symptoms due to impaired reuptake [54]. In this context, an SCFA‐enriched metabotype could represent a metabolic ‘on‐switch’ that amplifies symptoms in already vulnerable individuals; however, this remains a hypothesis at this juncture.

Interestingly, the key metabolites defining the SCFA metabotype were positively correlated with IBS symptom severity and negatively correlated with WGTT, whereas in the Other metabotype the pattern was reversed, with key metabolites (including aldehydes and ketones) negatively correlated with symptom severity and positively correlated with transit time. The metabosignature model allows further identification of gradients in groups of metabolites which can often strongly relate to biological structure [55]. Among the three metabosignatures identified here, one signature showed a high proportion of SCFAs in agreement with the metabotype clustering; however, we also identified an additional signature with SCFAs present at lower abundance and mixed with BCFA, produced by fermentation of protein rather than carbohydrates [56]. Unlike the signature dominated by SCFAs, this mixed SCFA–BCFA signature did not correlate with WGTT or symptoms and was more abundant in the ‘Other' metabotype. These findings further emphasise that it is not merely the presence of specific metabolites, but the composition of the colonic microenvironment and the dominant microbial metabolic activity, that determines symptom expression in IBS.

This study has limitations. First, the sample size was relatively modest, and although the geographical spread of participants across the United Kingdom might reduce the impact of epiphenomenon, firm conclusions cannot yet be derived from our observations. Furthermore, the cross‐sectional design limits our ability to conclude causality, and the absence of controls restricts our ability to attribute observed VOC differences to IBS itself rather than to unrelated inter‐individual variability. It also means we cannot exclude the possibility that baseline VOC profiles may be similar between IBS and healthy individuals, and that the clinically meaningful distinction lies instead in patients' responses to these metabolites rather than in the metabolites themselves. The sample size further limits any pathophysiological insights, in that only 13 out of the 63 participants were randomised to ondansetron, thus limiting our ability to measure the impact of these novel metabolic subgroups on clinical response.

We acknowledge that both VOC and microbiome analyses are correlational, and the associations reported here cannot establish mechanistic pathways or causality. Further, we acknowledge that untargeted VOC analysis itself is an inherently broad screening approach that may be susceptible to residual confounding. Although the TRITON protocol standardised sample collection and processing, unmeasured factors such as diet, medications, stool water content and subtle site or batch effects could still influence VOC profiles, and it was not possible to fully adjust for these variables within the present dataset. The absence of precise dietary behaviour limits our ability to assert with confidence that the differences in SCFA abundance between metabotypes are not driven by dietary habits; however, the SCFA metabotype has also been identified at baseline in other studies where dietary habits were comparable between groups [22].

The strict inclusion criteria (all had IBS‐D, 75% of whom had severe symptoms) meant that few metabolites demonstrated a strong correlation pattern with stool form, likely reflecting the very narrow spectrum of scores returned for this metric during the index TRITON study (requiring a Bristol stool form scale result of five to seven for recruitment). Similarly, WGTT values were skewed towards the faster end of the spectrum, which likely limited our ability to detect relationships with other clinical or biological variables. Therefore, the absence of correlations with transit‐related measures may reflect study design rather than a true lack of effect. Still, the ability to derive two metabolically and phenotypically relevant groups from such a symptomatically homogenous group of IBS patients is promising. Finally, we acknowledge that SPME–GCMS analysis of the faecal headspace provides a narrower snapshot of metabolic activity compared with other analytical techniques, but it offers a rapid, non‐invasive and clinically scalable means of biologically sub‐classifying IBS patients, potentially helping to translate metabolic insights from bench back to bedside.

## 5 Conclusion

A faecal metabolic profile enriched in SCFAs and associated metabolites appears to be associated with features of a more severe IBS‐D phenotype, including pain, urgency, rapid transit and higher stool frequency. Causal inference cannot be made at this juncture given the cross‐sectional nature of our analysis and confirmation in larger, prospective, high‐powered studies will be essential to clarify these relationships.

## Author Contributions

T.E.C., R.S. and C.S.P. planned the study. R.S. and A.C.F. were instrumental in conducted the initial clinical trial from which biological samples were collected. T.E.C., A.D., AM, F.H. and FW contributed to the analysis of the data and reported results. T.E.C., AD, AM, F.H. and FW generated figures and tables. T.E.C., A.D., F.H., F.J.W., R.S., D.M.P. and C.S.P. contributed to scientific discussions. T.E.C., FW and C.S.P. drafted the manuscript. T.E.C., AD, AM, F.H, F.J.W., A.C.F., R.S., D.M.P. and C.S.P. contributed to the revision of the manuscript. T.E.C., FW and C.S.P. verified the data and are responsible for the overall content as guarantors. All authors read and approved the final version of the manuscript.

## Funding

The authors have nothing to report.

## Conflicts of Interest

T.E.C. has received unrelated funding from GUTS UK and the British Society of Gastroenterology, as well as unrelated speaker and/or travel fees from Takeda and Ferring. F.J.W. has received grant support from the Biotechnology and Biological Sciences Research Council. R.S. has received grants from the National Institute for Health and Care Research, Sanofi GMB, Nestle and Vertex and has received unrelated consulting fees from Enterobiotix. The other authors declare no conflicts of interest.

## Supporting information


**Data S1:** apt70677‐sup‐0001‐Supinfo.docx.

## Data Availability

The data that support the findings of this study are available on request from the corresponding author. The data are not publicly available due to privacy or ethical restrictions.
