# Supplementary material for: The Emerging Short Chain Fatty Acid Enriched Metabotype in Irritable Bowel Syndrome and Its Potential Clinical Relevance
Source: Aliment Pharmacol Ther. 2026 Apr 17;64(2):208–21. doi: 10.1111/apt.70677 (PMC13309217; doi:10.1111/apt.70677)

**SUPPLEMENTAL MATERIALS**

**SM1: Recruitment sites**

SM1 – Recruitment site summary.

**SM2: Inclusion criteria**

| **Inclusion criteria** | **Exclusion criteria** |
| --- | --- |
| Age >18 years | Pregnancy or breast feeding |
| Rome IV IBS-D (moderate-severe) | Gastrectomy or Intestinal resection |
| Have completed standardised workup to exclude:  Microscopic colitis  Bile acid diarrhoea  Lactose intolerance  Coeliac disease | Other known organic gastrointestinal diseases (e.g., inflammatory bowel disease [Crohn’s disease, ulcerative colitis]) |
| Weekly average worst pain score ≥30 on a 0 to 100-point scale. | Unable or unwilling to stop restricted medication including regular loperamide, antispasmodics (e.g., hyoscine, mebeverine, peppermint oil, alverine citrate), eluxadoline, tricyclic antidepressant. |
| Stools with a consistency of 6 or 7 on the Bristol stool form scale for 2 or more days per week. | QTc interval ≥450ms for men or ≥ 470ms for women (assessed within the last 3 months by electrocardiogram (ECG)) |
|  | Previous chronic use of ondansetron, or contraindications to it |
|  | Patients who have started or altered dosing of selective serotonin re-uptake inhibitors or tricyclic antidepressants in the last 3 months, or who will change the dose during the trial |

**SM2 –** Inclusion criteria.

**SM3: Stool collection process**


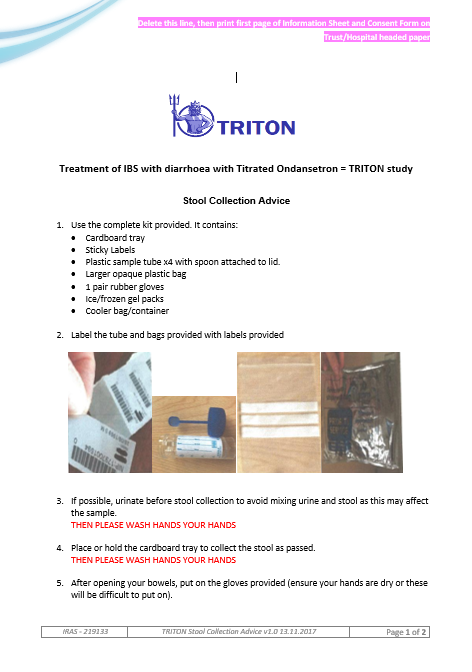


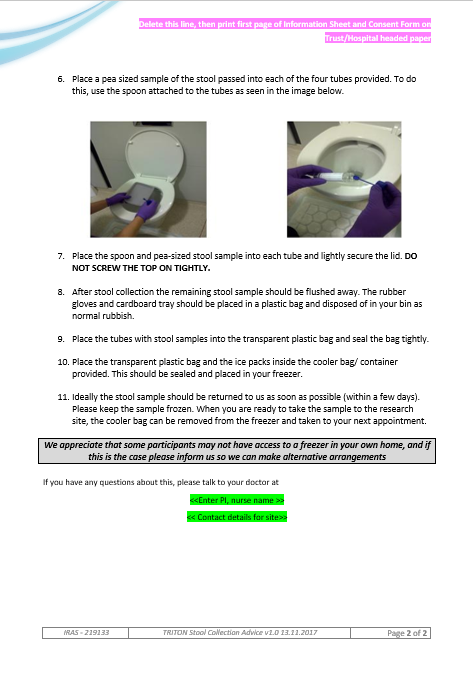


**SM4: Faecal volatile organic compound library**

| **Retention time (mins)** | **Faecal metabolites** |
| --- | --- |
| 7.10 | Ethanol |
| 7.70 | Propanal |
| 7.86 | Propan-2-one |
| 7.97 | Methylsulfanylmethane |
| 8.07 | Propan-2-ol |
| 8.25 | Carbon disulfide |
| 8.40 | Methyl acetate |
| 8.46 | Acetonitrile |
| 9.55 | 2-Methylpropanal |
| 10.10 | Propan-1-ol |
| 10.73 | Butanal |
| 10.81 | Butane-2,3-dione |
| 11.08 | Ethyl acetate |
| 11.09 | Butan-2-one |
| 11.35 | Butan-2-ol |
| 11.68 | Methyl propanoate |
| 11.71 | Chloroform |
| 12.51 | 2-Methylpropan-1-ol |
| 12.53 | 2-Methylpropane |
| 13.10 | 3-Methylbutanal |
| 13.36 | Acetic acid |
| 13.39 | 2-Methylbutanal |
| 13.83 | 3-Methylbut-3-en-2-one |
| 14.00 | Butan-1-ol |
| 14.19 | 1-Ethenylaziridine |
| 14.51 | Pentan-2-one |
| 14.55 | S-Methyl ethanethioate |
| 14.58 | 1-Sulfanylpropan-2-one |
| 14.63 | Ethyl propanoate |
| 14.72 | Pentanal |
| 14.72 | Pentane-2,3-dione |
| 14.83 | Propyl acetate |
| 15.14 | Methyl butanoate |
| 16.41 | Ethyl 2-methylpropanoate |
| 16.54 | 4-Methylpentan-2-one |
| 16.55 | (Methyldisulfanyl)methane |
| 16.67 | 3-Methylbutan-1-ol |
| 16.89 | Propanoic acid |
| 16.95 | 3-Methyl-2,3-dihydrofuran |
| 17.00 | Octane |
| 17.02 | Toluene |
| 17.10 | 3-Methylpentan-2-one |
| 17.23 | Methyl 2-methylbutanoate |
| 17.50 | 2-methylpent-1-en-3-one |
| 18.12 | Ethyl butanoate |
| 18.17 | Hexane-2,3-dione |
| 18.23 | Hexan-3-one |
| 18.49 | Propyl propanoate |
| 18.56 | Hexan-2-one |
| 18.73 | Hexane-3,4-dione |
| 18.74 | Butyl acetate |
| 18.84 | Hexanal |
| 19.04 | 2-Methylpropanoic acid |
| 19.14 | Methyl pentanoate |
| 20.01 | Ethyl 2-methylbutanoate |
| 20.13 | Butanoic acid |
| 20.15 | Heptan-3-one |
| 20.53 | 1-Cyclopropylpropan-1-one |
| 20.73 | 2-Methylpropyl propanoate |
| 20.78 | Ethylbenzene |
| 20.84 | Nonane |
| 21.25 | 5-Methylhexan-2-one |
| 21.38 | Furan-2-carbaldehyde |
| 21.49 | 3-Methyl-1H-indole |
| 21.56 | (E)-Hex-2-enal |
| 21.60 | 2-Methylhexan-3-one |
| 21.78 | Hexan-1-ol |
| 21.86 | Propyl butanoate |
| 21.94 | Ethyl pentanoate |
| 22.16 | 3-Methylbutanoic acid |
| 22.23 | Styrene |
| 22.24 | Butyl propanoate |
| 22.39 | 2-methylbutanoic acid |
| 22.46 | Heptan-2-one |
| 22.52 | Pentyl acetate |
| 22.66 | 3-Isothiocyanatoprop-1-ene |
| 22.77 | Heptanal |
| 22.88 | (3S)-3,7-Dimethylocta-1,6-diene |
| 22.91 | Methyl hexanoate |
| 23.02 | 2,6,6-Trimethylbicyclo[3.1.1]hept-2-ene |
| 23.42 | 3-(Methyldisulfanyl)prop-1-ene |
| 23.65 | Propyl 2-methylbutanoate |
| 23.66 | Pentanoic acid |
| 23.84 | S-Methyl 3-methylbutanethioate |
| 23.98 | 2-Methylpropyl butanoate |
| 24.11 | 3-Methylsulfanylpropanal |
| 24.22 | 2-Methyl-6-methylideneoct-2-ene |
| 24.35 | Methyl 2-methylpentanoate |
| 24.45 | (6E)-2,6-Dimethylocta-2,6-diene |
| 24.51 | Decane |
| 24.61 | 2,2,4,6,6-Pentamethylheptane |
| 24.69 | 4-Methyl-1-propan-2-ylbicyclo[3.1.0]hex-2-ene |
| 24.76 | 6,6-Dimethyl-2-methylidenebicyclo[3.1.1]heptane |
| 24.76 | 7-Methyl-3-methylideneocta-1,6-diene |
| 24.84 | 6-Methylheptan-2-one |
| 25.14 | 2-Pentylfuran |
| 25.26 | 5-Methylheptan-2-one |
| 25.27 | (2E,4E)-3,7-Dimethylocta-2,4-diene |
| 25.44 | Butyl butanoate |
| 25.47 | (Z)-Hept-2-enal |
| 25.53 | Ethyl hexanoate |
| 25.70 | 2-Methyl-5-propan-2-ylcyclohexa-1,3-diene |
| 25.79 | Octan-3-one |
| 25.85 | (Methyltrisulfanyl)methane |
| 25.95 | 4-Methylpentanoic acid |
| 26.00 | 6-Methylhept-5-en-2-one |
| 26.11 | Octan-2-one |
| 26.14 | Benzaldehyde |
| 26.45 | Octanal |
| 26.49 | Methyl heptanoate |
| 26.56 | (4R)-1-Methyl-4-prop-1-en-2-ylcyclohexene |
| 26.69 | 1-Methyl-4-propan-2-ylbenzene |
| 26.93 | Hexanoic acid |
| 27.02 | Butyl 2-methylbutanoate |
| 27.12 | 1,3,3-Trimethyl-2-oxabicyclo[2.2.2]octane |
| 27.16 | 1-Methoxy-4-methylbenzene |
| 27.16 | Butyl 3-methylbutanoate |
| 27.19 | 2-Methyl-4,5-dihydro-1,3-oxazole |
| 27.48 | 1-Methyl-4-propan-2-ylcyclohexa-1,4-diene |
| 27.93 | Undecane |
| 28.11 | 2,2,6-Trimethylcyclohexan-1-one |
| 28.46 | Methyl cyclohexanecarboxylate |
| 28.55 | Phenol |
| 28.71 | Thiolan-2-one |
| 28.81 | Propyl hexanoate |
| 28.90 | Ethyl heptanoate |
| 29.14 | 2-Phenylacetaldehyde |
| 29.22 | 3-(4-Methylpent-3-enyl)furan |
| 29.55 | Nonan-2-one |
| 29.72 | 3,7-Dimethylocta-1,6-dien-3-ol |
| 29.81 | Methyl octanoate |
| 29.89 | Nonanal |
| 29.90 | 1-Phenylethanone |
| 30.08 | Heptanoic acid |
| 30.08 | Heptanoic acid |
| 30.22 | Pentyl 2-methylbutanoate |
| 30.39 | Pentyl 3-methylbutanoate |
| 30.61 | 2-Methylpropyl hexanoate |
| 30.62 | 2-Methoxyphenol |
| 30.76 | Ethyl cyclohexanecarboxylate |
| 31.14 | Dodecane |
| 31.35 | 4-Methylphenol |
| 31.39 | Cyclohexa-1,4-diene |
| 31.90 | Hexyl butanoate |
| 31.91 | Butyl hexanoate |
| 32.03 | Ethyl octanoate |
| 32.04 | Cyclohexanecarboxylic acid |
| 32.13 | 1-Phenylpropan-2-one |
| 32.66 | 6-Methyloxan-2-one |
| 32.67 | 1,3,4-Oxadiazole |
| 32.69 | Decan-2-one |
| 32.72 | 4-Methyl-1-propan-2-ylcyclohex-3-en-1-ol |
| 32.81 | 1-Methyl-4-isopropyl-cyclohexyl 2-hydroperfluorobutanoate |
| 32.84 | Methyl 2-phenylacetate |
| 32.98 | Octanoic acid |
| 33.05 | Decanal |
| 33.34 | 2-(4-Methylcyclohex-3-en-1-yl)propan-2-ol |
| 33.73 | Methyl 2-hydroxybenzoate |
| 33.87 | Propyl cyclohexanecarboxylate |
| 33.91 | 1,3-Ditert-butylbenzene |
| 34.19 | 4-Ethylphenol |
| 34.80 | Hexyl pentanoate |
| 34.93 | Undecan-4-one |
| 35.41 | 2-Methyl-5-prop-1-en-2-ylcyclohex-2-en-1-one |
| 35.47 | 3-Propan-2-ylbenzaldehyde |
| 35.56 | Piperidin-2-one |
| 35.71 | Undecan-2-one |
| 35.76 | Methyl 3-phenylpropanoate |
| 35.98 | 1-Methoxy-4-[(E)-prop-1-enyl]benzene |
| 36.09 | 3-Ethyl-4-methylpyrrole-2,5-dione |
| 36.51 | 2,3,5-Trimethyl-4-methylidenecyclopent-2-en-1-one |
| 36.51 | 4-Ethenyl-4-methyl-1-propan-2-yl-3-prop-1-en-2-ylcyclohexene |
| 36.83 | (1R,5S,6R,7S,10R)-4,10-Dimethyl-7-propan-2-yltricyclo[4.4.0.01,5]dec-3-ene |
| 36.93 | Tetradecane |
| 37.30 | 2,4-Decadienal |
| 37.59 | 2-Methyl-5-(6-methylhept-5-en-2-yl)cyclohexa-1,3-diene |
| 39.01 | 1H-Indole |
| 40.94 | 1-Methyl-4-(6-methylhept-5-en-2-yl)benzene |

SM4 – Faecal volatile organic compound library.

**SM5: Self-Organizing Map**

To ensure metabotype allocation was robust, a Self-Organizing Map (SOM) was employed to validate the distinct metabolite profiles identified in the two groups. By applying the SOM to the unlabelled dataset, the algorithm was able to autonomously organize the data based on similarities in metabolite profiles. The successful separation of the two groups by the SOM, without prior labelling, confirmed the distinction of the two metabotypes. This unsupervised approach provided robust evidence that the metabolite differences observed were inherent and not artifacts of initial group assignments.

The SOM was initialized with random weights, and during training, each input vector was compared to the weight vectors using Euclidean distance to identify the Best Matching Unit (BMU). The weights of the BMU and its neighbours were updated based on a Gaussian neighbourhood function. This process iteratively refined the SOM until stability of the model was achieved, allowing for effective visualization and clustering of the data into distinct metabotypes.


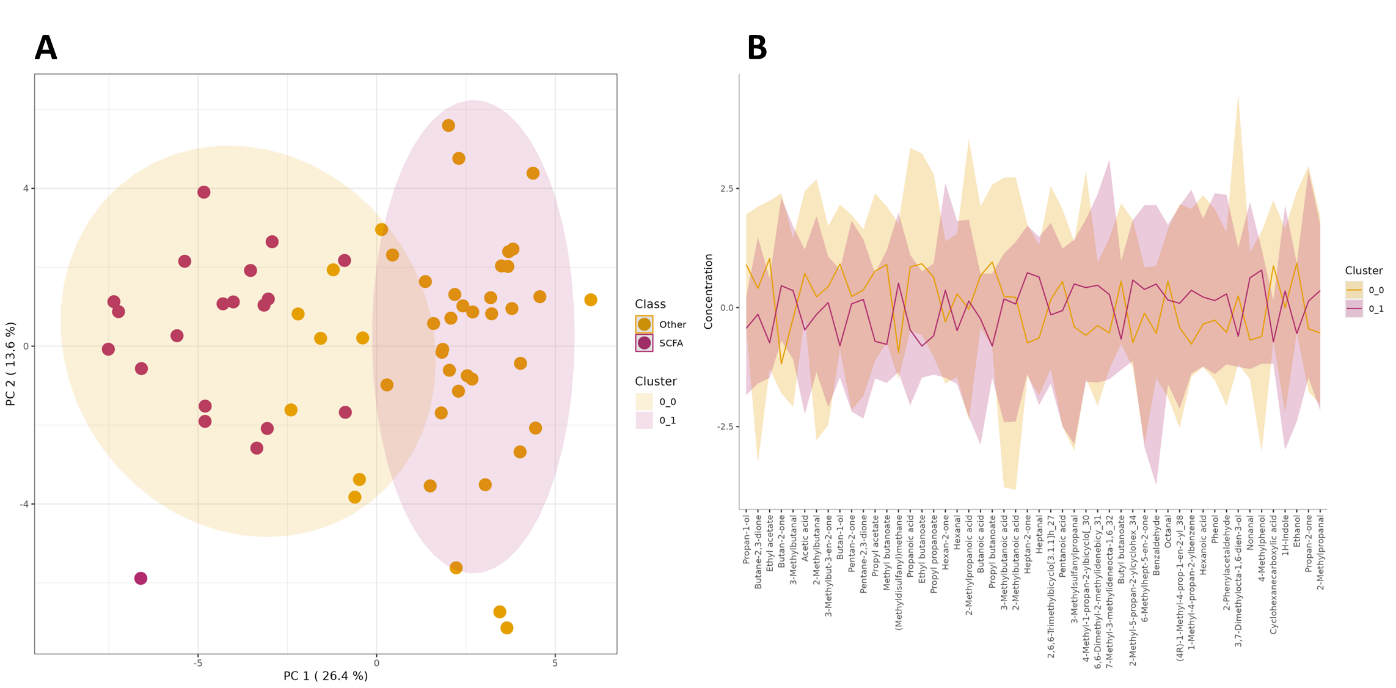


SM5 – Self organising map analysis. Left – Two groups (ovals) demonstrating the ability to discriminatory between the two metabotypes with accuracy based on unsupervised analysis of metabolite profiles within the entire dataset (0_0 = SCFA metabotype; 0_1 = Other metabotype). Right – Metabolite composition of two clusters identified by unsupervised SOM analysis demonstrating concordance with the changes seen on univariate analysis – Metabolites on x axis, Relative ionic abundance on Y axis (0_0 = SCFA metabotype; 0_1 = Other metabotype). (PC – principal component).

**SM6: Metabolite-symptom correlations**

|  | **SCFA metabotype** | | | | | | | | | | | | | | | | **Other metabotype** | | | | | | | | | | | | | | |
| --- | --- | --- | --- | --- | --- | --- | --- | --- | --- | --- | --- | --- | --- | --- | --- | --- | --- | --- | --- | --- | --- | --- | --- | --- | --- | --- | --- | --- | --- | --- | --- |
|  | **SCFAs** | | | | **SCFA esters** | | | | | | | **Alcohols** | | | 3-7-Dimethylocta-1-6-dien-3-ol | Cyclohexanecarboxylic acid | (Methyldisulfanyl)methane | **Ketones** | | **Aldehydes** | | | | | | 4-Methylphenol | **Terpenes** | | | | 1H-Indole |
|  | Acetic acid | Propanoic acid | Butanoic acid | Pentanoic acid | Ethyl acetate | Propyl acetate | Propyl propanoate | Methyl butanoate | Ethyl butanoate | Propyl butanoate | Butyl butanoate | Ethanol | Propan-1-ol | Butan-1-ol |  |  |  | Butan-2-one | 6-Methylhept-5-en-2-one | 2-Methylpropanal | 3-Methylbutanal | Nonanal | 3-Methylsulfanylpropanal | Benzaldehyde | 2-Phenylacetaldehyde |  | 7-Methyl-3-methylideneocta-1,6-diene | (4R)-1-Methyl-4-prop-1-en-2-ylcyclohexene | 1-Methyl-4-propan-2-ylbenzene | 6,6-Dimethyl-2-methylidenebicyclo-3,1,1-heptane |  |
| IBS SSS | 0.125 | -0.017 | 0.122 | 0.160 | 0.075 | 0.018 | -0.040 | 0.181 | 0.093 | 0.090 | 0.123 | -0.053 | 0.036 | 0.105 | 0.034 | 0.131 | **-0.319*** | -0.198 | -0.129 | **-0.273** | -0.225 | -0.115 | **-0.274*** | -0.065 | **-0.305*** | 0.086 | 0.123 | -0.032 | -0.038 | -0.049 | -0.041 |
| Abdominal pain | 0.151 | 0.024 | 0.111 | -0.007 | 0.158 | 0.238 | 0.150 | **0.289*** | 0.221 | 0.240 | 0.171 | 0.136 | 0.178 | 0.196 | 0.022 | 0.254 | -0.182 | -0.149 | **-0.380*** | **-0.285*** | -0.176 | -0.258 | **-0.318*** | **-0.345*** | **-0.418*** | -0.173 | 0.009 | -0.087 | -0.018 | -0.051 | **-0.317*** |
| Urgency | **0.271*** | 0.161 | 0.170 | 0.067 | 0.173 | **0.304*** | 0.227 | **0.361*** | **0.264*** | **0.318*** | **0.279*** | 0.178 | 0.221 | 0.201 | 0.053 | **0.312*** | **-0.325*** | -0.057 | **-0.338*** | -0.235 | -0.192 | -0.049 | -0.218 | **-0.334*** | **-0.319*** | -0.226 | -0.103 | -0.149 | -0.155 | -0.112 | -0.221 |
| Stool frequency | 0.185 | 0.102 | 0.156 | -0.115 | 0.211 | 0.239 | 0.121 | **0.263*** | **0.292*** | **0.314*** | 0.203 | 0.174 | 0.170 | 0.169 | 0.053 | 0.237 | -0.060 | -0.177 | -0.153 | -0.235 | -0.210 | -0.196 | **-0.340*** | **-0.288*** | **-0.328*** | -0.220 | -0.009 | 0.083 | 0.150 | 0.153 | **-0.338*** |
| Loose stool days | 0.212 | 0.204 | 0.200 | 0.062 | 0.201 | **0.323*** | 0.257 | **0.323*** | 0.247 | 0.212 | 0.110 | **0.303*** | **0.305*** | 0.151 | 0.116 | **0.305*** | -0.019 | -0.143 | **-0.332*** | -0.198 | -0.168 | -0.098 | **-0.367*** | **-0.276*** | **-0.358*** | -0.178 | 0.163 | 0.023 | 0.068 | -0.018 | -0.090 |
| Stool consistency | 0.047 | -0.009 | 0.042 | -0.100 | -0.134 | -0.074 | 0.018 | 0.168 | 0.083 | 0.201 | 0.240 | -0.008 | -0.014 | 0.062 | -0.013 | 0.064 | 0.030 | 0.020 | 0.049 | -0.083 | -0.146 | -0.078 | -0.159 | -0.096 | -0.166 | -0.018 | 0.118 | **0.274*** | 0.192 | 0.208 | -0.118 |
| WGTT | -0.254 | **-0.265*** | -0.226 | -0.077 | **-0.282*** | **-0.313*** | **-0.275*** | **-0.320*** | **-0.273*** | **-0.289*** | -0.190 | **-0.304*** | **-0.396*** | -0.225 | -0.191 | **-0.283*** | **0.344*** | 0.176 | 0.013 | 0.225 | 0.215 | 0.111 | 0.228 | -0.149 | 0.187 | **0.297*** | 0.111 | 0.123 | 0.209 | **0.281*** | -0.173 |
| Anxiety | 0.007 | -0.117 | 0.071 | -0.065 | -0.111 | -0.011 | -0.067 | 0.146 | -0.056 | 0.046 | 0.024 | -0.042 | -0.019 | -0.040 | -0.075 | -0.104 | 0.089 | 0.000 | -0.192 | -0.147 | -0.014 | -0.082 | **-0.324*** | -0.229 | -0.239 | -0.084 | 0.008 | 0.084 | 0.097 | 0.052 | -0.169 |
| Depression | 0.162 | 0.084 | 0.141 | 0.046 | 0.152 | 0.150 | 0.020 | 0.092 | 0.070 | 0.053 | 0.061 | 0.110 | 0.128 | -0.031 | -0.039 | 0.144 | 0.011 | 0.010 | **-0.267*** | -0.059 | -0.012 | -0.032 | -0.184 | -0.185 | -0.112 | -0.074 | -0.184 | -0.158 | 0.048 | -0.041 | -0.093 |

SM6 – Correlation plot demonstrating the pattern of Pearson correlation coefficients between all 50 volatile organic compounds included in the analysis and the IBS metrics measured.


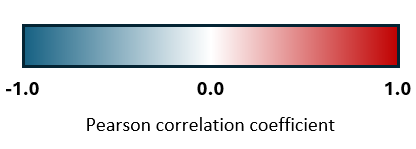

Supplement: Supplementary file 1 — Data S1: apt70677‐sup‐0001‐Supinfo.docx. [file APT-64-208-s001.docx]
